# A two-phase spatiotemporal chaos-based protocol for data integrity in IoT

**DOI:** 10.1038/s41598-024-58914-x

**Published:** 2024-04-15

**Authors:** Mimouna Abdullah Alkhonaini, Farhan A. Alenizi, Yahia Hasan Jazyah, Sangkeum Lee

**Affiliations:** 1 Department of Computer Science, College of Computer and Information Sciences, Prince Sultan University, Riyadh, Saudi Arabia; 2 Electrical Engineering Department, College of Engineering, Prince Sattam Bin Abdulaziz University, Al-Kharj, 11942 Saudi Arabia; 3 Faculty of Computer Studies, Arab Open University, Ardiya, Kuwait; 4 Department of Computer Engineering, Hanbat National University, Daejeon, 34158 South Korea

**Keywords:** Integrity ensuring, Internet of Things, Chaos theory, Network security, Secure data transmission, Computational science, Computer science, Information technology

## Abstract

One of the biggest problems with Internet of Things (IoT) applications in the real world is ensuring data integrity. This problem becomes increasingly significant as IoT expands quickly across a variety of industries. This study presents a brand-new data integrity methodology for Internet of Things applications. The “sequence sharing” and “data exchange” stages of the suggested protocol are divided into two parts. During the first phase, each pair of nodes uses a new chaotic model for securely exchanging their identity information to generate a common sequence. This phase’s objectives include user authentication and timing calculations for the second phase of the recommended method’s packet validation phase. The recommended approach was tested in numerous settings, and various analyses were taken into account to guarantee its effectiveness. Also, the results were compared with the conventional data integrity control protocol of IoT. According to the results, the proposed method is an efficient and cost-effective integrity-ensuring mechanism with eliminates the need for third-party auditors and leads to reducing energy consumption and packet overhead. The results also show that the suggested approach is safe against a variety of threats and may be used as a successful integrity control mechanism in practical applications.

## Introduction

To build the Internet of Things (IoT) technology, RFIDs, wireless sensor networks (WSN), and other intelligent devices are used, together with middleware, web-based software platforms, and a reliance on cloud computing^1^. In IoT, a huge amount of data is generated for storing and processing which results in various challenges^2^. Flexibility and high efficiency in this architecture have made the IoT suitable for use in various fields^3^. One of the most basic requirements in most Internet of Things application scenarios is to ensure no unwanted and unauthorized changes in the huge amount of data produced by it. This requirement is called data integrity^4^. Data integrity is more important in some IoT application scenarios (such as smart health networks). In the smart health network, sensors and objects in the network often exchange patients’ vital signs and emergency data (such as heart rate, occurrence of epileptic seizures, occurrence of an emergency event in medical centers, etc.). In most cases, the smallest changes in this data may cause irreparable loss of life and money^5^. In addition, the nature of the IoT and the large amount of data produced in it has caused the need to pay attention to data integrity more than ever. Because, on the one hand, the data exchanged by things is of great variety (images, textual data, voice, etc.)^6^, and on the other hand, most of these data are unstructured^7,8^. Controlling the integrity of this large amount of diverse and unstructured data will be essential to guarantee the network’s performance and reliability. Therefore, so far, several techniques have been presented to provide data integrity in IoT. The review of recent research shows that we will face at least one of the following challenges in using these methods in IoT^9^:

- Some of these techniques are not effective for use in real environments due to their limitation of application.
- The high computational complexity of some of these techniques makes them difficult to use in real-time scenarios.
- The need for central control or a Third-Party Auditor (TPA) to ensure data integrity in some methods makes these techniques ineffective in some operational scenarios of the IoT.

Metadata and hashing techniques are often limited to use in the file domain^10^. On the other hand, encryption-based techniques have high computational complexity and are not effective for equipment with limited computing power. Two other common techniques for controlling data integrity are digital signatures and blockchain. Digital signature techniques perform integrity control through a central controller. In this situation, if the data distortion has been done by a man-in-the-middle attack and just before the data is delivered to the receiver node, the mentioned technique will not be able to identify the lack of data integrity. On the other hand, the methods based on chain blocks, although they can acceptably control data integrity, their use will require acceptance of delay and high computational load. On the other hand, most integrity assurance methods use a TPA system to control integrity and assume that this system is fully protected. In fact, in these solutions, the issue of integrity ensuring is dependent on the security of the TPAs.

These problems and limitations have caused the need to provide a method for ensuring data integrity in the IoT to be felt more than ever. Therefore, providing a solution to solve the mentioned problems is a research priority in the field of IoT. In this paper, an efficient solution to provide data integrity in IoT is presented. To guarantee the security of information communication between objects, this technique makes use of a hash function for random permutation based on a brand-new two-way chaotic model. First, a unique two-way chaotic model for random permutation is presented in this study. This model is more effective than other chaotic models that have been provided in the past. Second, the strategy put out in this article may be applied to any IoT architecture or circumstance. The suggested algorithm’s performance is independent of the kind of data being transmitted.

### Data integrity in IoT

This subsection focuses on the concept of data integrity and its challenges within the context of the Internet of Things (IoT).

In an IoT system, data integrity ensures that data remains unmodified and unaltered throughout its lifecycle, encompassing collection, transmission, storage, and processing. This means the data received is identical to the data originally sent by the sensor or device. Maintaining data integrity is crucial for reliable decision-making and accurate operation within the network.

Challenges of data integrity in IoT include:

- Limited resource devices: many IoT devices have constrained processing power and battery life. Complex data integrity solutions requiring high computational resources may not be feasible for these devices.
- Real-time processing: certain applications demand real-time data processing. Computationally expensive integrity methods can introduce unacceptable delays that hinder real-time functionality.
- Decentralized architectures: IoT networks may not always have a central authority to manage data integrity. Techniques relying on third-party auditors (TPAs) become inapplicable in such decentralized scenarios.
- Data variety: the diverse nature of data exchanged in IoT (images, text, sensor readings) necessitates integrity solutions that can effectively handle various data types.

Understanding these challenges is crucial for developing efficient data integrity solutions tailored to the specific needs of IoT environments. The following sections of the paper will delve into existing data integrity techniques and propose a novel approach that addresses these challenges.

The remainder of the essay is structured as follows: the related works are discussed in section two. The suggested approach will be given in the third section, and its effectiveness will be assessed from various angles in the fourth section. The conclusions are made in the fifth section, which is the last.

## Related works

In^6^, a data integrity control method with low energy consumption for IoT applications is presented. This technique uses a linear chaotic map to produce a pseudo-random permutation of data and control their integrity. The sequence sharing phase and the data exchange phase make up this approach. A linear chaotic sequence is generated as a result of exchanging the model’s starting parameters between the two systems during the sequence-sharing phase. This chaotic map has a pseudo-random pattern. In the data exchange phase, the data block is swapped using the linear chaotic map by the sending node. After the data is received, the inverse pattern of the linear chaotic map is used to recover the data and detect potential tampering. It should be noted that according to^10^, the linear chaotic map—which is used in this article—has some shortcomings such as limited key space and low efficiency against differential and Brute-Force attacks.

In^11^, an integrity control algorithm in IoT based on digital signature is presented. This algorithm uses a digital signature with ZSS format to reduce the computational overhead in the integrity control process. This process includes four calculation steps. In the first step, the parameters of the system are initialized by a TPA system. Then, private and public keys are created based on the system parameters, and in the third step, the private key and data content are used to generate a digital signature. Finally, the integrity control operation will be performed based on the public key and the exchanged message.

An Industrial IoT (IIoT) Threat Intelligence Integrity Audit (TIIA) method based on blockchain is put out in^12^. The double chain structure is used in this method. Also offered is a simple technology-based audit system for building an entire attack chain. This scheme’s audit chain employs a quick method for deleting unnecessary blocks. In^13^, a distributed edge computing based on blockchain for real-time data integrity control in IoT environments is proposed. This architecture can eliminate the requirement for centralized TPA servers using the edge computing framework. Also, this scheme supports a blockchain-based edge computing IoT design for improving the security and scalability of data integrity. However, the use of this combination, in addition to increasing the implementation cost, results in high computational complexity and significant overhead.

Research^14^ proposes a hierarchical framework for guaranteeing data integrity in IoT devices. This hierarchical approach employs a fast integrity verification for current data, followed, if required, by a low overhead secure technique for current and historical data integrity verification. A maritime transportation system (MTS) integrity verification technique for IoT-enabled is provided in^15^. In^16^ a method for ensuring data integrity in IoT environments is presented. Using digital signatures for public-key cryptography, this method focuses on assuring the integrity of data. This method involves applying the Elliptical Curve Digital Signature (ECDS) to a predefined Software Defined Networking (SDN) architecture. In IoT environments, ECDS offers the same level of security as RSA with fewer keys.

Instead of focusing on just monitoring and controlling collected data, research^17^ introduces a blockchain-based system to ensure the data from IoT devices is tamper-proof. This system minimizes data loss and keeps verification costs low through grouped information and location-based synchronization. Research^18^ creates a new way to send data safely in wireless mesh networks used for the Internet of Things (IoT). It builds secure channels for data to travel between devices and verifies the data isn’t tampered with. The researchers tested their method using a computer simulation and found it improves data delivery and reduces errors.

Current cloud-based systems for managing IoT data in smart cities lack transparency and raise trust concerns. Research^19^ proposes a new approach using a “Blockchain-of-Blockchains” (BoBs) system. This decentralized system ensures both the security of the data and compatibility between the different blockchain networks used by various organizations within a smart city. Keeping data secure in the cloud is crucial for IoT systems. Traditional methods rely on a trusted third party to verify data integrity, which isn’t always reliable. Research^20^ introduces a new approach that uses advanced cryptography (Lifted EC-ElGamal cryptosystem and bilinear pairing) along with blockchain technology. This eliminates the need for a trusted third party and allows for efficient verification while also protecting the privacy of the data within the IoT system.

Research^21^ tackles the problem of faulty data by creating an online method to monitor data integrity. This method can catch various data errors, like wrong formats, timing issues, and incorrect values. The researchers tested their method on motion tracking sensors and showed it significantly improves data quality. Research^22^ introduces the Trusted Consortium Blockchain (TCB) framework, a novel approach to address data integrity challenges in smart manufacturing’s big data landscape. TCB harnesses the power of blockchain technology to guarantee the security and verifiability of the massive datasets generated by the multitude of devices and stakeholders interacting within the manufacturing process. The framework leverages the Hyperledger Fabric Modular platform to achieve high transaction throughput and minimal latency, all while upholding robust data integrity. Table 1 summarizes the studied works.

**Table 1:** Summary of the literature review.

Ref	Year	Methodology	Limitations
^6^	2018	Linear chaotic map for pseudo-random permutation of data	Limited key space, low efficiency against differential and brute-force attacks^10^
^11^	2019	Digital signature with ZSS format to reduce computational overhead	Requires Trusted Third Party (TPA) system for initialization
^12^	2022	Blockchain-based Threat Intelligence Integrity Audit (TIIA) with double chain structure	High implementation cost, complex auditing
^13^	2021	Distributed edge computing with blockchain for real-time data integrity	High computational complexity, significant overhead
^14^	2021	Hierarchical framework for fast and secure data integrity verification	Not specified
^15^	2021	Data integrity checking with original data recovery for maritime IoT	Focused on a specific application domain
^16^	2022	Elliptical Curve Digital Signature (ECDS) for public-key cryptography	Relies on centralized SDN architecture
^17^	2021	Blockchain for tamper-proof data and low verification cost	Not specified
^18^	2020	Secure channels for data transmission in wireless mesh networks	Focused on data transmission security, not general data integrity
^19^	2022	Blockchain-of-Blockchains (BoBs) for interoperable data integrity in smart cities	Increased complexity compared to single blockchain
^20^	2020	Blockchain with advanced cryptography (Lifted EC-ElGamal cryptosystem) for privacy-preserving data integrity	Complex cryptographic techniques
^21^	2020	Online data integrity monitoring method for digital sensors	Focused on sensor data integrity, not general IoT data
^22^	2023	Trusted Consortium Blockchain (TCB) for big data integrity in smart manufacturing	Requires consortium formation and management

## Proposed model

This section will outline the suggested protocol for ensuring data integrity in the Internet of Things, which is based on the two-way chaotic model. The suggested approach is based on the integrity control model described in^6^ and seeks to address two of this model’s drawbacks: Improving the random permutation process using a two-way chaotic model. The chaos map used in^6^ faces the limitation of the key space and the sensitivity of the key is very low. The key space used in^6^ only covers the interval (0,1). This method does not examine the security of the used chaos model. The modeling of this chaotic map through the analysis of the patterns in chaotic sequences will be very simple. Also, the set of computational operations used in this method to generate a pseudo-random sequence based on chaos imposes a significant computational burden on the processor. For this reason, in the proposed method, a new chaotic model with extended key space and higher sensitivity will be used compared to^6^. This technique can significantly improve the security level of the proposed protocol and at the same time significantly reduce the computational energy consumption.

Usability of the proposed model for various network configurations. The protocol proposed in^6^ can be used in a specific configuration in IoT. This protocol considers the existence of a server (as a TPA system) as its configuration requirements. In the proposed method, this requirement is removed and the proposed model can be used to control the integrity of various communication patterns and network configurations. In this way, the generalization of the application will be one of the advantages of the proposed method, which makes it suitable for various applications.

In the following, we will first explain the system model and the assumptions considered in the proposed model, and then we will present the two-way chaotic model used in the proposed method. Finally, the proposed data integrity control procedure will be presented.

### System model and assumptions

The proposed protocol operates within a network model consisting of three primary components:

1. IoT devices: these are resource-constrained devices equipped with sensors and actuators that collect and transmit data. They communicate directly with each other or via the data server depending on the network configuration.
2. Data server (optional): this server acts as a central repository for storing data generated by IoT devices. Its presence is not mandatory, and devices can exchange data through multi-hop paths in decentralized configurations. In any scenario, data integrity checks are performed by the communicating parties during data exchange.
3. Communication medium (Internet): the internet facilitates communication between IoT devices and the data server (if present). Security measures are crucial here due to the open nature of the internet.

The assumptions considered in the current research are as follows:

- Device deployment: IoT devices are deployed in open environments without dedicated physical protection, making them vulnerable to physical tampering.
- Data server security: the data server (if present) is assumed to be physically secure, preventing unauthorized access.
- Resource constraints: IoT devices have limited processing power and energy resources. The data server, however, is considered to have no such limitations.

Also, this research considers attackers who may target various network components:

- Network attackers: these attackers aim to compromise network components like IoT devices, routers, or communication links.
- Eavesdroppers: these attackers can intercept all network traffic, allowing them to resend previously exchanged messages, impersonate other nodes, or inject malicious data.
- Data disruption: the attacker’s objective is to alter data related to IoT devices, potentially disrupting network performance.

Building upon these assumptions and the attack model, this research proposes a novel data integrity model that safeguards data within the IoT network. The details of the proposed method will be explained in the following section. In the following, we will explain the steps of the proposed method. Table 2, lists the notations used in this paper.

**Table 2:** List of the notations.

Symbol	Description
${x}_{n}$	The n-th item of the chaotic sequence
$\alpha$	The spatial key of the spatiotemporal chaotic model
$\beta$	The temporal key of the spatiotemporal chaotic model
⊕	the bitwise XOR operation
IX_i_	ID for IoT device i
ϕ()	hash function
\	concatenation operator
C(M,k)	the encryption of message M using key k
K_A,B_	The communication key between items A and B
PIX_A_	The alias identity of node A
CH_A_	The time-varying bit string of node A for generating secret key
R_A_, R_B_, R_c_	The random seeds used to generate chaotic sequences
Seq	A sequence containing validation periods

### Two-way chaotic model

Compared to linear chaos systems, two-way chaos has a much more complex behavior and presents more characteristics. Dual chaotic systems are usually modeled through partial differential equations, mixed differential equations, or Coupled Map Lattices (CML). In the proposed algorithm using CML, a data permutation method based on a two-way chaotic model is provided.

#### The proposed two-way chaotic map

The Nonlinear Chaotic Algorithm (NCA) is generated based on the logistic map. A logistic map can be defined by the following equation ^23^:

1 $$x_{n + 1} = \mu x_{n} \left( {1 - x_{n} } \right),\; n = 1,2,3, \ldots$$

where $0<\mu \le 4 ,\;{x}_{n}\epsilon (\mathrm{0,1})$. In the above equation, if $3.57\le \mu \le 4$, the logistic map will show a chaotic behavior. One of the disadvantages of using this model is the limited space of the key and, as a result, its low security. Therefore, in this paper, a NCA based on logistic map is presented. This model is shown in the following equation:

2 $$x_{n + 1} = \frac{{\left( {1 - \frac{1}{{\beta^{2} }}} \right) \cdot \left[ {\left( {1 - x_{n} } \right)\left( {1 + \beta } \right)} \right]^{\beta } \cdot \cot \left( {\frac{\alpha }{1 + \beta }} \right) \cdot \tan \left( {\alpha x_{n} } \right)}}{{\beta^{\beta } }},\;n = 1,2,3, \ldots \mathop {\lim }\limits_{x \to \infty }$$

where $\alpha \in \left(\mathrm{1.5,1.57}\right],\;\beta \in [\mathrm{3,10}]$ are the keys of this chaotic model and, ${x}_{n}\epsilon \left(\mathrm{0,1}\right)$ refers to the *n*th element of the chaotic sequence.

**Figure 1:**
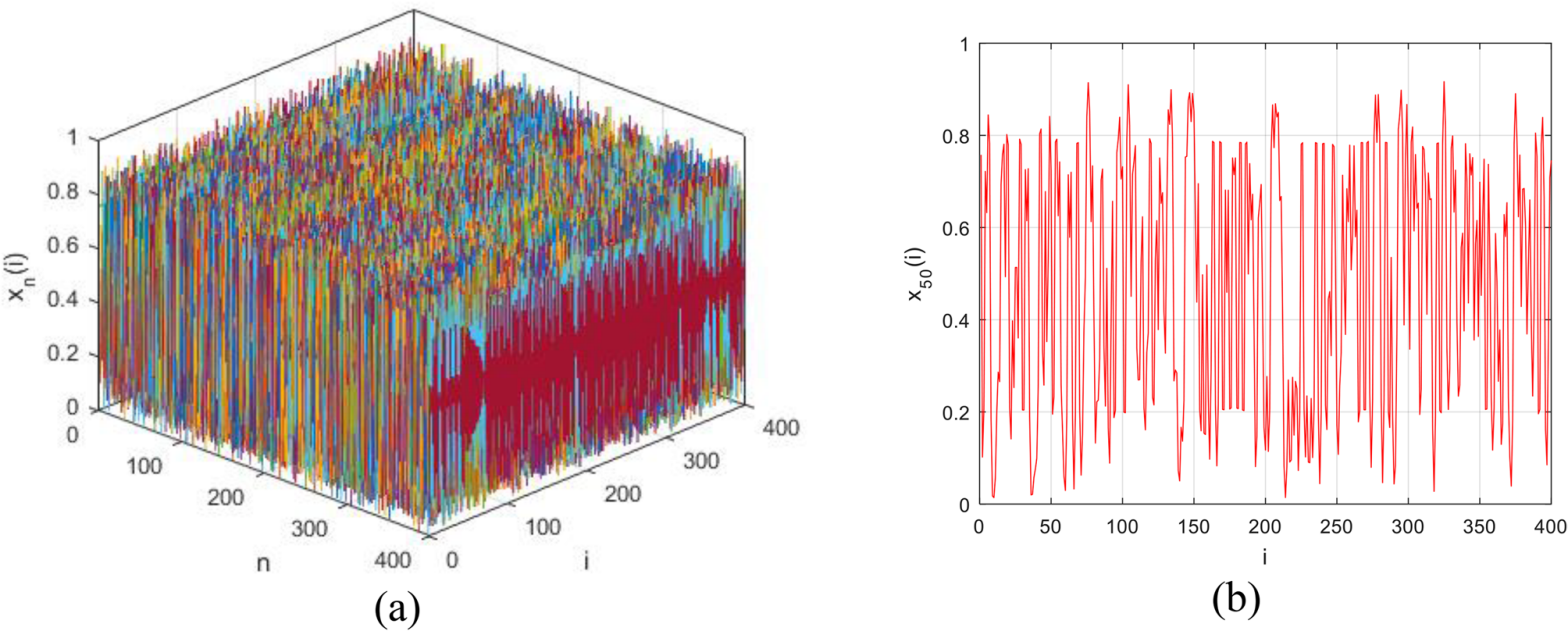
Proposed chaotic model (**a**) the attractor of the proposed NCA-based CML (**b**) chaotic sequence for n = 50.

CML is a model of a dynamic system with discrete space and position, which has successive states, and is often used as a primary model to study dynamics in two-way chaotic systems. A two-way CML system can be modeled as the following equation:

3 $$\left\{ {\begin{array}{*{20}l} {x_{n + 1} = \left( {1 - \varepsilon } \right)f\left( {x_{n} \left( i \right)} \right) + \frac{\varepsilon }{2}\left\{ {f\left( {x_{n} \left( {i - 1} \right)} \right) + f\left( {x_{n} \left( {i + 1} \right)} \right)} \right\} } \hfill \\ {f\left( x \right) = \mu x\left( {1 - x} \right)} \hfill \\ \end{array} } \right.$$

where *i* refers to the spatial index, *n* is usually called the temporal index and ε is the constant of composition and is in the interval (0,1). Also, $3.57\le \mu \le 4,\;0<x<1 ,\;0<f\left(x\right)<1$ defines the range of values that can be used in Eq. (3). To take advantage of the NCA, Eq. (2) can be replaced in Eq. (3). By doing this, a two-way chaotic model is obtained as follows:

4 $$\left\{ {\begin{array}{*{20}l} {x_{n + 1} = \left( {1 - \varepsilon } \right)f\left( {x_{n} \left( i \right)} \right) + \frac{\varepsilon }{2}\left\{ {f\left( {x_{n} \left( {i - 1} \right)} \right) + f\left( {x_{n} \left( {i + 1} \right)} \right)} \right\} } \hfill \\ {f\left( x \right) = \frac{{\left( {1 - \frac{1}{{\beta^{2} }}} \right) \cdot \left[ {\left( {1 - x_{n} } \right)\left( {1 + \beta } \right)} \right]^{\beta } \cdot \cot \left( {\frac{\alpha }{1 + \beta }} \right) \cdot \tan \left( {\alpha x_{n} } \right)}}{{\beta^{\beta } }} } \hfill \\ \end{array} } \right.$$

where $\varepsilon \in \left(\mathrm{0,1}\right)$ and ${x}_{n}\left(i\right)\in \left(\mathrm{0,1}\right)$. The rest of the values in the above relation are similar to relation 2. Figure (1-a) illustrates the attractor of the NCA-based CML presented in relation (4) for $0<i\le 400,\;0<n\le 400,\;\varepsilon =0.1,\;\alpha =1.57,\;\beta =3.5$. Also, Fig. (1-b) illustrates a chaotic sequence of this CML, while the temporal index is considered as *n* = 50.

As it is clear from the figure, this chaos model can provide suitable and non-linear characteristics in the range of zero to one. As a result, it will be suitable for use as a hash function in the proposed method.

#### Hashing and producing chaotic permutation in the proposed method

The pseudo-random pattern of the two-way chaotic sequence can be used to design an efficient hash function. In this section, we will describe this function and how to generate chaotic permutation. In the following, we show the content of the input data to be hashed as *M*. Before starting the process and regardless of the data type *M*, we convert it into a one-dimensional array as $M=\{{m}_{1},{m}_{2},\dots ,{m}_{n}\}$. In this array, m_i_ represents the *i*-th byte of this data. The proposed method shuffles the contents of the packets at the byte level and includes the following processing steps:

##### Key generation

In vector *M*, we convert all values of the data sequence to decimal. This makes each byte of data be described as a number between 0 and 255. Then calculate the sum of the numerical values and then divide the sum successively by the number 10, so that the result of this division is obtained as a number in the interval [0,1]. For example, if the total is equal to 1,819,127; the result of successive division will be equal to 0.1819127. This normalized number will be used as the initial value of *x*_*1*_ in Eq. (4) to generate the chaotic sequence. We define this pseudo-random chaotic sequence as $A=\{{a}_{1},{a}_{2},\dots ,{a}_{n}\}$.

##### Permutation

We sort the values of the sequence *A* in descending order so that the order of sorting the values of this sequence is obtained as permutation sequence *IX*. We call this sequence as chaotic permutation and it will be used independently in the sampling process of data (“Hashing and Producing chaotic permutation in the proposed method” section). Sequence *IX* shows the pattern of movements for the members of chaotic Sequence *A*. For example, *IX(i)* shows which element in *A* is replaced by the *i*-th element in the sorted sequence. By using the order of sequence *IX* and applying the same permutation pattern to sequence *M*, the permuted sequence $M^{\prime}=\left\{{m}_{IX(1)},{m}_{IX(2)},\dots ,{m}_{IX(n)}\right\}$ will be achieved.

##### Diffusion

Based on the sequence *A* obtained from Eq. (4), we calculate the sequence $Y=\left\{{y}_{i}|i=\mathrm{1,2},\dots ,n\right\}$ using the following equation:

5 $$y_{i} = \left\lfloor {\left( {a_{i} \times 10^{14} } \right) \;mod256} \right\rfloor$$

The above equation shows that the values of *y* are always between 0 and 255. Then, using this vector, we can diffuse data $M^{\prime}$ through the following equation:

6 $$c_{i} = m_{i} \oplus y_{i}$$

In the above equation, the ⊕ operator indicates the bitwise XOR operation. The result of these steps will be the hashed data *c*.

### Ensuring data integrity in IoT by proposed method

In the rest of this section, the proposed protocol will be described to identify data tampering in the IoT. For this purpose, we will mention the signs and symbols used first. We will use the symbols IXi, ϕ() and \ to display the IoT device ID, hash function and concatenation operator, respectively. Also, C(M,k) denotes the encryption of message M using key k and $\oplus$ denotes the bitwise XOR operation. The symbols K_A,B_ are used to represent the communication key between two pairs of items, such as A and B. In the proposed model, IoT devices do not need to store the encryption key in their memory; Instead, each device stores a time-varying bit string as CH in its memory. When necessary, a device like IX_A_ may create the secret key for communication with a device like IX_B_ as C(CH_A_\CH_B_, CH_A_\CH_B_). In this instance, employing this bit string prevents an attacker from obtaining the secret key. Having this property protects items against physical assault and impersonation. Each gadget also communicates using an alias rather than its true identity. This alias identity, which is displayed as *PIX*_*A*_ for the device with *IX*_*A*_ ID, will be created using the hash function as $\varphi (I{X}_{A}\backslash k)$. Considering that the secret key of communication is unique for both network devices; therefore, the alias identity to communicate between each pair of devices will be unique. This feature will make it impossible for attackers to identify the original identity of the device.

During the process of sending data and at pseudo-random time points, each network device sends validation packets to the receiving device to check this information and ensure the integrity of all previously sent data which are as follows:

1. Sequence sharing
2. Data transfer

In the following, we will explain each of these steps.

#### Sequence sharing phase

This two-way exchange phase will be used to create a pseudo-random sequence between the two communication parties. If we consider *IX*_*A*_ and *IX*_*B*_ as transmitter and receiver nodes, respectively; then the sequence-sharing phase in the proposed protocol will be done through the following steps:

1. In the first step, the *IX*_*A*_ object calculates its secret key *k*_*A,B*_ and *PIX*_*A*_ alias identity based on the previously described process, and encrypts it as C(R_A_,k_A,B_) by generating a random number such as *R*_*A*_. Also, by using the hash function, the result of concatenation of the secret key, alias identity and random number is converted into $\mathrm{\varphi }(PI{X}_{A}\backslash {k}_{A,B}\backslash {R}_{A})$ and all this information is sent to *IX*_*B*_ in the form of the first message. The hash function is used in this message to guarantee both the message’s and the data’s integrity. As a result, the message structure will be in form of $\langle PI{X}_{A},C\left({R}_{A},{k}_{A,B}\right),\mathrm{\varphi }(PI{X}_{A}\backslash {k}_{A,B}\backslash {R}_{A})\rangle$.
2. The *IX*_*B*_ object obtains the *R*_*A*_ random number using its calculated secret key. We show the random number calculated by this node as ${R}_{A}^{\prime}$. Then, in order to ensure the integrity of the received data, using the calculated random number, *IX*_*B*_ calculates the value of $\mathrm{\varphi }(PI{X}_{A}\backslash {k}_{A,B}\backslash {R}_{A}^{\prime})$ and compares its value with the value received in the first message. If $\mathrm{\varphi }\left(PI{X}_{A}\backslash {k}_{A,B}\backslash {R}_{A}^{\prime}\right)\ne \mathrm{\varphi }(PI{X}_{A}\backslash {k}_{A,B}\backslash {R}_{A})$, then *IX*_*B*_ rejects the sequence sharing request. The communication parties, use the value of *S* to generate a pseudo-random sequence of integers. Also, the sequence generator value can be periodically updated to ensure accuracy. Then the *IX*_*B*_ object sends the second message of the sequence sharing phase to *IX*_*A*_. This message contains the encrypted values of *PIX*_*A*_ alias identity, random number *R*_*B*_, generator *S* and random number *R*_*A*_, along with the output of hashing and concatenation of all these values with the secret key. Thus, this message will have the structure of $\langle C\left({\{PI{X}_{A},R}_{B},S,{R}_{A}\},{k}_{A,B}\right),\mathrm{\varphi }(PI{X}_{A}\backslash {k}_{A,B}\backslash {R}_{A}\backslash S\backslash {R}_{B})\rangle$.
3. The node *IX*_*A*_ calculates the random number *R*_*B*_ using the key ${k}_{A,B}$ and by comparing $\mathrm{\varphi }\left(PI{X}_{A}\backslash {k}_{A,B}\backslash {R}_{A}\backslash S\backslash {R}_{B}\right)\ne \mathrm{\varphi }(PI{X}_{A}\backslash {k}_{A,B}\backslash {R}_{A}\backslash S\backslash {R}_{B}^{\prime})$ confirms the authenticity of the received message. If the verification is successful, then *IX*_*A*_ stores the generator sequence *S* in its memory and decrements the value of *R*_*B*_ by one unit and sends it in the form of a message as $\langle C\left({\{PI{X}_{A},R}_{A},{R}_{B}-1\},{k}_{A,B}\right),\mathrm{\varphi }(PI{X}_{A}\backslash {k}_{A,B}\backslash {R}_{A}\backslash {R}_{B}-1)\rangle$ to confirm the received value.
4. The IX_B_ object verifies the validity of the received message, and if it is confirmed, the sequence-sharing phase is terminated.

**Figure 2:**
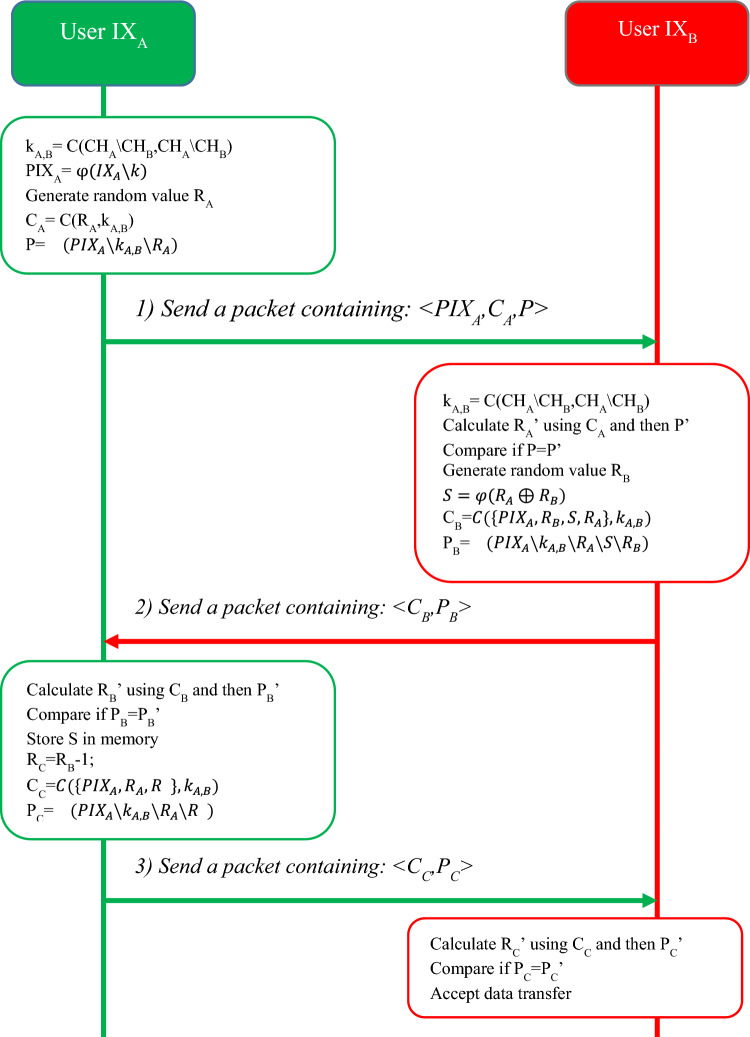
Diagram of sequence sharing phase in the proposed method.

The steps of the sequence sharing phase in the proposed method are shown in Fig. 2.

After this stage is completed, two objects, each of which has a secret sequence generator similar to S, produce a sequence using the chaotic model. The data transmission stage may be completed after completing this.

#### Data transfer phase

**Figure 3:**
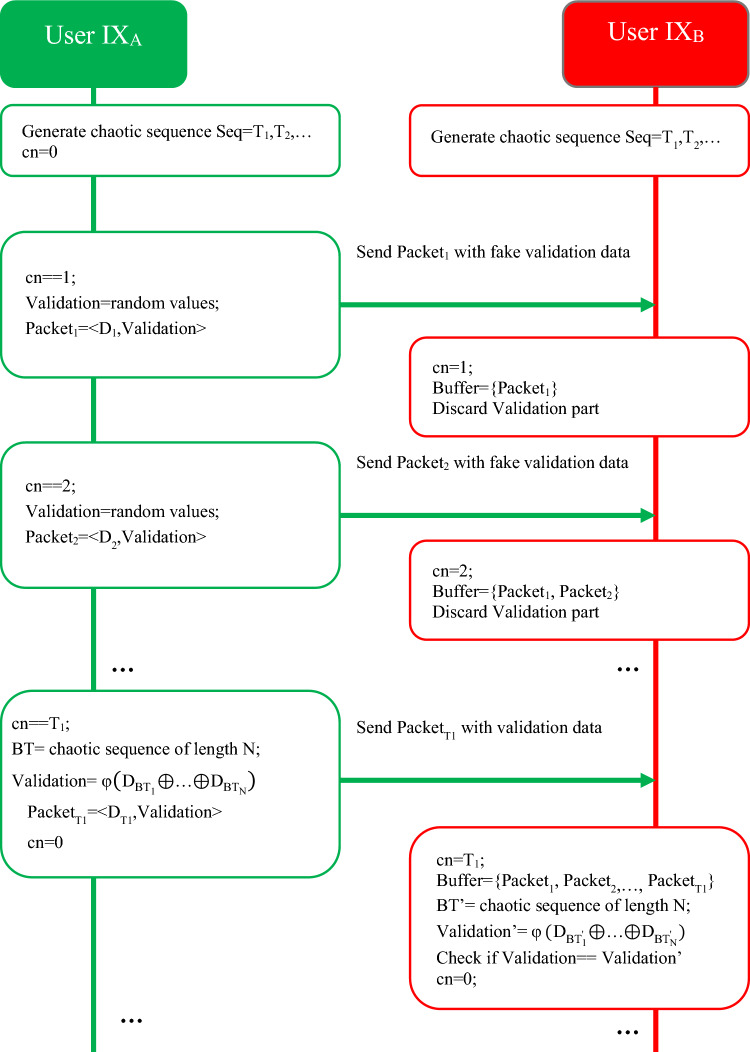
Diagram of the data transfer phase in the proposed method.

The communication parties will have a pseudo-random sequence such as *Seq*, which is generated based on the chaotic sequence. In the following, we consider the created sequence as a set of periods such as $Seq={T}_{1},{T}_{2},\dots$. This sequence will be used to hide the authentication information of the data exchange parties. For this purpose, *IX*_*A*_ and *IX*_*B*_ nodes create a counter for the order of exchanged packets such as *cn*, and by sending each data packet from *IX*_*A*_ to *IX*_*B*_, both objects increase their counter by one unit. The node IXA injects validation information into this packet when the cn counter reaches the first element of the Seq sequence, i.e. T1, and sets the counter to cn = 0 in both objects. The suggested method’s data transmission phase procedures are shown as a flowchart in Fig. 3. In this way, normal messages contain fake validation information; While the validation messages will be the result of hashing the previously sent messages $\varphi (B)$. In this case, an attacker will not be able to recognize the exchange of packets containing validation information, and as a result, it will not be possible for the attacker to distinguish between normal and validation packets. In the proposed method, the sequence values of *Seq* are determined in the interval^10,20^.

The number of packets will be merged into the validation message) from previously unauthenticated and sent data packets. Then, using the XOR operator and the hash function, it hashes the selected packets. For example, if the set of previously unauthenticated batches is BT = B_1_,B_2_,…,B_T1_ and the generated chaotic permutation (for *N* = 3) is *I* = {2,5,1}; then, Validation content will be $V=\varphi ({B}_{2}\oplus {B}_{5}\oplus {B}_{1})$. This validation packet is sent to the *IX*_*B*_. The node IX_B_ produces the chaotic permutation of length *N* again and by searching for these unauthenticated confirmation batches in its buffer memory; it calculates the validation content as ${{\text{V}}}^{\prime}$. If $V={V}^{\prime}$, then all previously unauthenticated confirmation messages will be confirmed, and otherwise, this whole set will be rejected.

## Simulation and results

This section comprehensively evaluates the effectiveness of the proposed two-phase spatiotemporal chaos-based protocol for data integrity in IoT from four key perspectives:

1. Resistance of the proposed protocol against attacks: in this test, the performance of the proposed method will be examined in the face of various types of network attacks. For this purpose, it should be possible to simulate different scenarios in the presence of attackers. ProVerif (PV) tool has been used to assess the proposed protocol’s resilience to different assaults. This simulation tool may use algebraic processes to construct a variety of attack scenarios and analyze the suggested protocol from many security angles.
2. Computational efficiency evaluation: computational complexity is a critical factor for any security protocol, especially for resource-constrained IoT devices. This evaluation investigates the computational overhead imposed on IoT device processors by the proposed protocol. The experiment measured the processing resources required for integrity control operations under the proposed method. A high-performance computing platform was used to simulate a large number of devices and various data sizes. The measurements were compared to existing data integrity protocols to assess the relative efficiency of the proposed approach. Lower computational complexity translates to faster processing times and improved device responsiveness within the IoT network.
3. Analysis of communication overhead: data integrity protocols often introduce additional data packets for security purposes. This analysis investigates the communication overhead associated with the proposed protocol. The experiment measured the increase in packet volume compared to a baseline scenario without integrity control. The simulation environment was configured to represent a realistic network scenario with a specific number of devices and message transmission rates. Additionally, a comparison was made with existing protocols to evaluate the relative overhead introduced by each approach. Minimizing communication overhead is essential for maintaining network performance and scalability in resource-constrained IoT environments.
4. Evaluation of energy efficiency: it is caused by two main components: the energy consumption resulting from the execution of computing operations and the energy consumption resulting from data transmission. In an integrity control protocol with low computational complexity, the number of processor computational operations will be reduced and as a result, the computational energy consumption will be reduced. On the other hand, reducing the communication overhead in a protocol will also reduce the packet size, which results in a reduction in the energy consumed for data transmission. In this test, the two components of energy consumption will be evaluated according to different parameters. To accurately evaluate these values, the MATLAB simulation tool, which is a high-performance numerical calculation software, will be used.

In the rest of this section, we will present the results of the tests and analyses.

### Protocol simulation

This evaluation assesses the protocol’s resilience against various network attacks. A comprehensive simulation environment was created to emulate diverse attack scenarios. The PV tool, known for its ability to construct attack scenarios using formal methods, was employed for this purpose. The protocol was subjected to a series of simulations, including common attacks like man-in-the-middle (MitM), replay attacks, and message modification attempts. The results were analyzed to determine the protocol’s ability to withstand these attacks. This analysis provides valuable insights into the protocol’s security posture under various threat models. PV uses an algebraic process to define protocols and simulate exchanges to prove some security features. The final state of simulating the performance of a protocol by PV are: “Successful provident of a feature” or “Discovery of an attack”. A security feature has been successfully proved if the security protocol has passed the simulation procedure and been able to demonstrate the claimed security feature. The two sides of the communication will then be connected in a variety of random ways. The assertions of the communication parties have been compared in order to demonstrate the mutual and effective authentication between them, and the key’s secrecy has also been assessed. Any prospective or deterministic attack on security protocols may be found with the PV simulator.

### Computational efficiency

Assuming fixed block encryption, it can be deduced that the complexity of encryption and hash operations is O(N), where N is the size of a message. Nevertheless, the permutation sequence generation will have a computational cost of O (n log(n)), where n is the number of data blocks in each validation message. These complexity estimates allow us to deduce that the computational difficulty of the key sharing stage in the proposed technique is O(N), and that the computational complexity of the data exchange phase for validation packages is O(N + n log(n)) and that it is O(1) for regular packets. As previously noted, 10 ≤ n ≤ 20 is found to be the suitable amount of data batches in each validation message. As a consequence, the computational cost of the suggested solution will be quite low. The suggested technique, however, is less computationally demanding than the protocol now in use since we know that n ≪ N.

### Energy efficiency

Energy efficiency is a paramount concern in IoT deployments. This section investigates the energy consumption of the proposed protocol from two perspectives:

- Computational energy consumption: the number of processor operations required for integrity control directly impacts energy usage. This experiment measured the computational complexity of the proposed protocol by simulating various message lengths and device configurations. The results were compared to existing methods to assess the relative energy consumption for integrity control tasks. Lower computational complexity translates to reduced energy consumption due to fewer processor cycles.
- Data transmission energy consumption: the communication overhead, as analyzed in this Section, also influences energy consumption. This experiment evaluated the impact of the protocol’s packet size on data transmission energy usage. The simulation environment considered different network topologies and transmission distances to reflect real-world scenarios. Smaller packet sizes typically require less energy to transmit. The combined effects of computational and transmission energy consumption were then assessed to determine the overall energy efficiency of the proposed protocol.

In this experiment, a network consisting of 100 devices that reflect the characteristics of the MICA 2 node is considered in an environment with dimensions of $100\times 100$ meters. During the simulation process, each of these 100 nodes will send 100 data packets with a transmission rate of 19.2 kb/s, and the amount of energy consumed from these transmissions will be calculated. Consequently, the values reported in this section will be the result of an exchange of 10,000 packets (100 packets sent by each of the 100 nodes). Also, in these tests, the efficiency of the proposed protocol will be compared with the methods presented in^6^ and^12^ and the existing conventional approach to ensure data integrity. In the conventional approach, a MAC will be sent along with each packet to control data integrity. This approach has been used as a comparison criterion in most previous researches, and for this reason, this protocol will be used in the proposed method to compare energy efficiency.

In the first scenario, we will consider the size of the MAC in the traditional integrity control protocol, the size of the block in the method^12^ and the size of the permutation sequence in the proposed method and^6^. In this case, the energy consumption resulting from data transmission for the proposed method and the conventional integrity control protocol will be almost the same. Because the size of the packets is the same in both protocols. Nevertheless, in the proposed method, the permutation sequence is calculated only for validation packets; while the conventional integrity control protocol, constructs MAC for all packets. As a result, it is natural that in the case of using the proposed protocol, the processor consumes less energy than the conventional integrity control protocol. On the other hand, the proposed method has performed data integrity through less computational operations than^6^, the result of which will be a reduction in processor energy consumption. These results can be seen in Table 3.

**Table 3:** Comparison of energy consumption of the proposed protocol with previous methods in scenario 1.

	MAC size (bits)	Proposed (µJ)	Aman et al.^6^ (µJ)	Zhang et al.^12^ (µJ)	Conventional (µJ)	Improvement vs Ref.^6^ (%)	Improvement vs Ref.^12^ (%)	Improvement vs Conv. (%)
Processor energy consumption	64	390.9751	497.7793	863.5556	844.495	21.46	54.72	53.70
	128	424.3790	533.0095	1268.2366	1320.998	20.38	66.54	67.87
	192	457.9214	571.0850	1424.7778	1548.705	19.82	67.86	70.43
	256	491.0953	602.6839	1962.8095	2093.597	18.52	74.98	76.54
Radio energy consumption	64	5479.456	5481.692	6018.125	5483.452	≈ 0.00	8.95	≈ 0.00
	128	6019.528	6022.193	6394.481	6019.128	≈ 0.00	5.86	≈ 0.00
	192	6586.506	6588.237	6994.212	6590.915	≈ 0.00	5.83	≈ 0.00
	256	7164.916	7165.909	7672.856	7168.275	≈ 0.00	6.62	≈ 0.00

**Figure 4:**
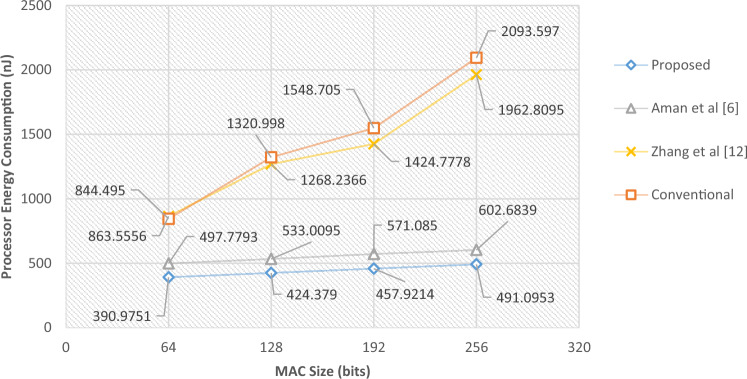
Amounts of computing energy consumed in scenario 1.

**Figure 5:**
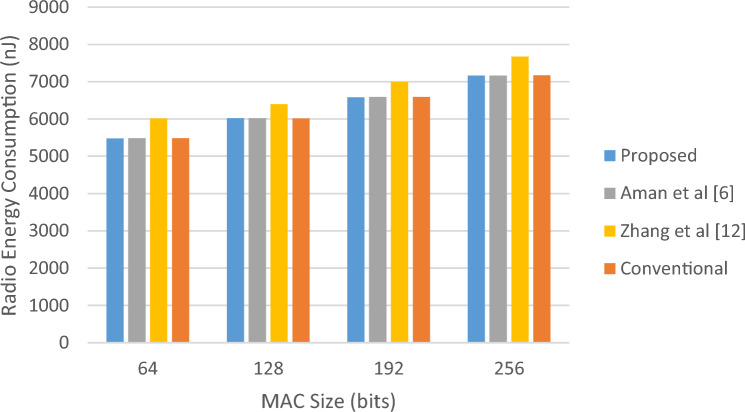
Amounts of radio energy consumed in scenario 1.

According to Table 3, with the increase in the size of the MAC/permutation sequence, the difference in the energy consumption of the processor in the proposed method and the compared methods also increases. In such a way that for the permutation MAC/sequence with the length of 64 bits, the proposed method saves 53.7% of the processor energy and for the length of 256 bits, this amount of saving reaches more than 76.54%. The amounts of energy consumed due to processing and data transmission for this experiment are shown as diagrams in Figs. 4 and 5, respectively.

The complexity of the pseudo-random permutation model built on the two-way chaotic model, as previously mentioned in the preceding section, is what gives the proposed protocol its security. It will be increasingly challenging to identify the permutation pattern as the chaotic sequence becomes shorter. Therefore, the suggested protocol may be thought of as working with a 64-bit hashing algorithm^6^. To provide the same degree of security, most MAC-based protocols need MACs greater than 128 bits. With these justifications, we will assume the new protocol’s permutation sequence size is 64 bits in the second scenario and the traditional protocol’s MAC size is changed from 64 to 128 bits. In this situation, the suggested protocol would deliver packets of a lesser size than the standard protocol by lengthening the MAC. It follows that in this situation, the suggested solution may likewise lower radio energy use. Table 4 displays these outcomes.

**Table 4:** Comparison of energy consumption of the proposed protocol with previous methods in scenario 2.

	MAC size (bits)	Proposed (µJ)	Aman et al.^6^ (µJ)	Zhang et al.^12^ (µJ)	Conventional (µJ)	Improvement vs Ref.^6^ (%)	Improvement vs Ref.^12^ (%)	Improvement vs Conv. (%)
Processor energy consumption	64	390.9751	497.7793	863.5556	844.495	21.46	54.72	53.70
	128	390.9751	497.7793	863.5556	1320.998	21.46	54.72	68.31
	192	390.9751	497.7793	863.5556	1548.705	21.46	54.72	71.04
	256	390.9751	497.7793	863.5556	2093.597	21.46	54.72	77.39
Radio energy consumption	64	5479.456	5481.692	6018.125	5483.452	≈ 0.00	8.95	≈ 0.00
	128	5479.456	5481.692	6018.125	6019.128	≈ 0.00	8.95	8.97
	192	5479.456	5481.692	6018.125	6590.915	≈ 0.00	8.95	16.86
	256	5479.456	5481.692	6018.125	7168.275	≈ 0.00	8.95	23.56

**Figure 6:**
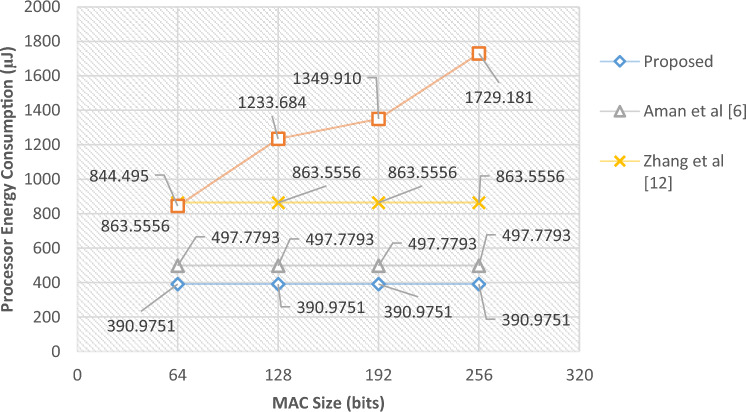
Amounts of computing energy consumed in scenario 2.

**Figure 7:**
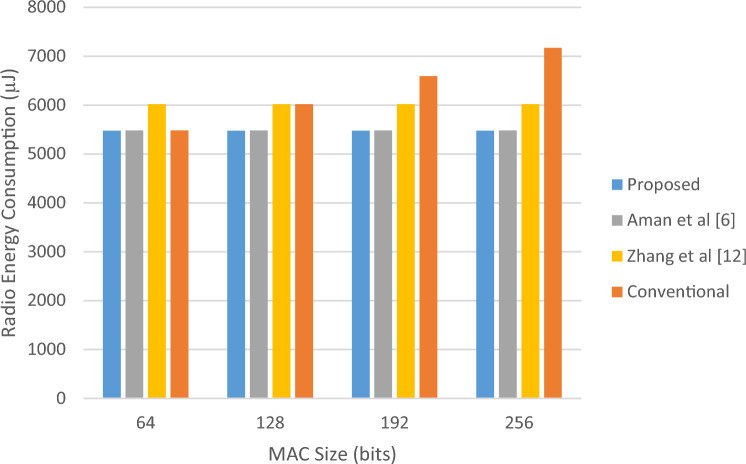
Amounts of radio energy consumed in scenario 2.

Based on the findings in Table 4, it is possible for the suggested protocol to lessen radio transmission energy consumption by more than 23% in addition to saving processing energy. In Figs. 6 and 7, these findings are shown as diagrams.

**Figure 8:**
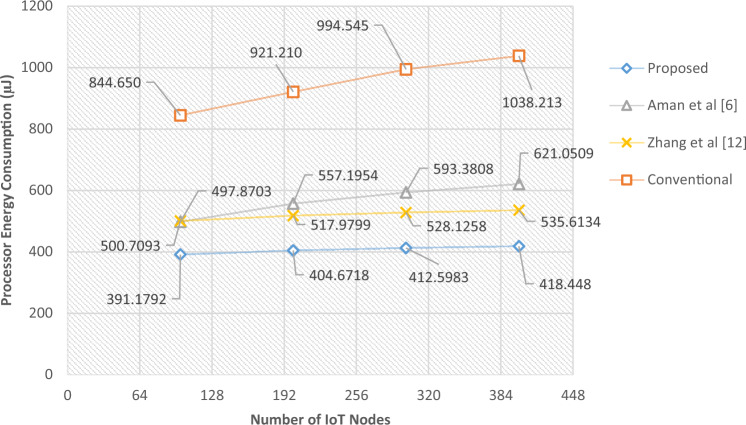
Amounts of computational energy consumed per number of nodes.

**Figure 9:**
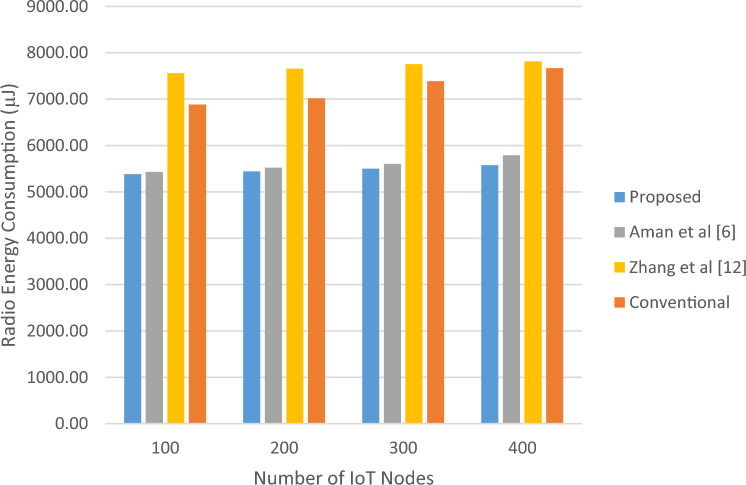
Amounts of radio energy consumption per number of nodes.

The energy usage of the suggested technique under various IoT node count scenarios will be examined in the sections that follow. In this experiment, the number of active network devices is increased from 100 to 400 nodes, and the length of the sequence generated by the chaotic model (or MAC size) is taken to be equal to 64 bits. Then, for these changes, the average values of consumed radio and computational energy for each packet have been calculated. The results of this test are shown in Table 5. These results are shown in Figs. 8 and 9.

**Table 5:** Comparison of the energy consumption of the proposed protocol with the previous methods in terms of changes in the number of nodes.

	Number of IoT Nodes	Proposed (µJ)	Aman et al.^6^ (µJ)	Zhang et al.^12^ (µJ)	Conventional (µJ)	Improvement vs Ref.^6^ (%)	Improvement vs Ref.^12^ (%)	Improvement vs Conv. (%)
Processor energy consumption	100	391.1792	497.8703	500.7093	844.650	21.43	21.87	53.69
	200	404.6718	557.1954	517.9799	921.210	27.37	21.87	56.07
	300	412.5983	593.3808	528.1258	994.545	30.47	21.87	58.51
	400	418.4480	621.0509	535.6134	1038.213	32.62	21.87	59.70
Radio energy consumption	100	5380.40	5429.20	7562.60	6883.452	0.898843	28.86	21.84
	200	5439.80	5521.60	7656.30	7019.128	1.481455	28.95	22.50
	300	5499.90	5601.60	7752.70	7390.915	1.815553	29.06	25.59
	400	5576.40	5792.00	7812.30	7668.275	3.722376	28.62	27.28

Based on the results obtained in this experiment, the proposed method can reduce energy consumption in all cases, both in terms of data transmission and computation.

### Communication overhead

Communication overhead is a crucial factor in resource-constrained IoT environments, as excessive data packets can strain network bandwidth and device battery life. This analysis investigates the communication overhead introduced by the proposed two-phase spatiotemporal chaos-based protocol compared to existing data integrity solutions.

Traditional cryptographic techniques, such as RSA, often incur a communication overhead of 128 to 256 bytes due to the large key sizes involved. Additionally, implementing message authentication codes (MAC) for data integrity adds further overhead. MAC algorithms typically require a key size of 128 bits, translating to at least 8 bytes of overhead per packet. Some MAC-based techniques might even require a larger MAC size (16 bytes) for enhanced security, further increasing the communication cost.

The proposed protocol aims to minimize communication overhead by employing a more lightweight approach. Here’s a breakdown of the overhead components:

- Chaotic sequence exchange: the initial exchange of chaotic sequences for key generation in the first phase might introduce some overhead. However, this exchange only occurs once when two devices establish communication. The size of the chaotic sequence can be optimized to balance security and communication efficiency.
- Hash function usage: the protocol utilizes hash functions for generating aliases and potentially for message authentication. The specific hash function chosen and its output size will determine the overhead associated with this operation. Lightweight hash functions can be employed to minimize this overhead.
- Validation packets: during data transmission, the protocol transmits validation packets at pseudo-random intervals to ensure data integrity. The size and frequency of these packets can be carefully designed to achieve a balance between security and communication efficiency.

To accurately assess the communication overhead of the proposed protocol, a comprehensive simulation was conducted. Due to the 64-bit hashing function, the proposed protocol will produce only 8 bytes of overhead per packet. On the other hand, if we also consider sending the MAC along with each packet, at least 8 bytes of overhead will be produced in each packet. Therefore, the suggested protocol has reduced communication overhead than current approaches as a consequence.

### The security of the proposed chaotic model

Using important sensitivity and information entropy criteria, the efficacy of the proposed two-way chaotic model is examined in this section. Two different kinds of picture and text data are used as input in these studies, and the results are compared to the chaotic model shown in^6^. The first criterion evaluated in this section is information entropy. Entropy is considered as one of the key criteria in measuring the randomness of information. This criterion is calculated by the following equation^24^:

7 $${\text{H}}\left( {\text{m}} \right){ = } - \mathop \sum \limits_{i = 1}^{{2^{n} - 1}} p\left( {m_{i} } \right)\log_{2} \frac{1}{{p\left( {m_{i} } \right)}}$$

where *m* represents the data (image or text) and $p({m}_{i})$ represents the probability of occurrence of the value ${m}_{i}$ in the data *m*. In data that is encoded by bytes (such as image pixels or each character of text), an ideal random state will have an entropy value of 8. Therefore, a close-to-ideal hashed data will have an entropy close to 8.

The meaning of key sensitivity is the smallest amount of change in the keys of the chaotic sequence, which can be used to perform hashing correctly. Based on the evaluations, the smallest significant change for α and β keys in the proposed method is equal to ${10}^{-15}$ and the least significant change for ε key in the proposed two-way chaotic model is equal to ${10}^{-17}$. Thus, the sensitivity of the key in the proposed method will be at least equal to ${10}^{-15}\times {10}^{-15}\times {10}^{-17}={10}^{-47}$. This high sensitivity in the proposed method can be seen as the result of using the two-way chaotic model. This is while the method presented in^6^ uses only one key with the valid range of $(\mathrm{0,1})$ and the sensitivity of this key is equal to 10^–11^. The results of the security evaluation of the proposed chaotic model and the comparison of its efficiency with the method presented in^6^ are shown in Table 6.

**Table 6:** Comparison of entropy and key sensitivity of the proposed chaotic model with previous method.

	Criterion	Proposed	Aman et al.^6^
Image data	Entropy	7.9974	7.6315
	Key sensitivity	10^–47^	10^–11^
Textual data	Entropy	7.9831	7.6150
	Key sensitivity	10^–47^	10^–11^

These results show that the proposed method, in addition to advantages such as reducing energy consumption and computational overhead; it can provide a higher level of security for detection of data tampering in the IoT.

## Conclusion

The proposed method uses a permutation sequence and hash function based on the two-way chaotic model to control data integrity. Studying the performance of the proposed protocol in the simulation process showed that the proposed method is safe against a wide range of attacks, and in addition, it can reduce the overhead of data being exchanged in the network. On the other hand, the low computational complexity of the proposed method makes this protocol prevent the loss of computing resources and reduce the energy consumption of the processor significantly. All these features make the proposed method a suitable and reliable solution to control data integrity in real-time applications.

In the future works, it will be tried to provide a secure and energy efficient routing protocol for use in the IoT by combining the proposed model with a routing protocol. Also, studying the application of the proposed method in other types of wireless networks can be the subject of future research.

## Data Availability

All data **generated** or analyzed during this study are included in this published article.
